# Sex-specific Mendelian randomisation to assess the causality of sex differences in the effects of risk factors and treatment: spotlight on hypertension

**DOI:** 10.1038/s41371-023-00821-1

**Published:** 2023-04-07

**Authors:** Sophie C. de Ruiter, A. Floriaan Schmidt, Diederick E. Grobbee, Hester M. den Ruijter, Sanne A. E. Peters

**Affiliations:** 1 Julius Center for Health Sciences and Primary Care, University Medical Center Utrecht, Utrecht University, Utrecht, the Netherlands; 2 Department of Cardiology, Amsterdam University Medical Centers, University of Amsterdam, Amsterdam, the Netherlands; 3 Department of Cardiology, Division Heart and Lungs, University Medical Center Utrecht, Utrecht University, Utrecht, the Netherlands; 4 Institute of Cardiovascular Science, Faculty of Population Health, University College London, London, UK; 5 UCL British Heart Foundation Research Accelerator Centre, London, UK; 6 Laboratory of Experimental Cardiology, Division Heart and Lungs, University Medical Center Utrecht, Utrecht University, Utrecht, the Netherlands; 7 The George Institute for Global Health, School of Public Health, Imperial College London, London, UK

**Keywords:** Risk factors, Genetics research

## Abstract

Hypertension is a key modifiable risk factor for cardiovascular disease. Several observational studies have found a stronger association of blood pressure and cardiovascular disease risk in women compared to men. Since observational studies can be affected by sex-specific residual confounding and reverse causation, it remains unclear whether these differences reflect actual differential effects. Other study designs are needed to uncover the causality of sex differences in the strength of risk factor and treatment effects. Mendelian randomisation (MR) uses genetic variants as instrumental variables to provide evidence about putative causal relations between risk factors and outcomes. By exploiting the random allocation of genes at gamete forming, MR is unaffected by confounding and results in more reliable causal effect estimates. In this review, we discuss why and how sex-specific MR and *cis*-MR could be used to study sex differences in risk factor and drug target effects. Sex-specific MR can be helpful to strengthen causal inferences in the field of sex differences, where it is often challenging to distinguish nature from nurture. The challenge of sex-specific (drug target) MR lays in leveraging robust genetic instruments from sex-specific GWAS studies which are not commonly available. Knowledge on sex-specific causal effects of hypertension, or other risk factors, could improve clinical practice and health policies by tailoring interventions based on personalised risk. Drug target MR can help to determine the anticipated on-target effects of a drug compound and to identify targets to pursue in drug development.

## Introduction

Cardiovascular disease (CVD) is the most common cause of death in both men and women. In 2019, an estimated 18.6 million people died of CVD, which equates to 31% of all deaths in men and 35% of all deaths in women [1]. While the lifetime risk of developing CVD is similar between women and men, women tend to present their first cardiovascular event at a later age than men [2]. Women are also more likely to experience stroke, which occurs at a later age, while coronary heart disease is the most common type of CVD in men [1].

Hypertension, defined as having systolic blood pressure (SBP) ≥ 140 mmHg, or diastolic blood pressure (DBP) ≥ 90 mmHg, is an important modifiable risk factor for CVD [1]. The Non-Communicable Disease Risk Factor Collaboration estimated that 626 million women and 652 million men had hypertension globally in 2019 [3]. SBP levels above 115 mmHg accounted for an estimated 8.5 million deaths worldwide in 2015 [4]. In general, women have lower levels of blood pressure than men, especially at younger age [5, 6]. A study on 30,372 individuals from eight cohorts in the UK found a maximum sex difference at age 26, where the average SBP was 8.2 mmHg higher in men than in women [7]. After this age, the average SBP in women showed a steeper rise than in men, resulting in a smaller sex difference at higher ages.

### Sex differences in the association between hypertension and CVD

**Fig. 1:**
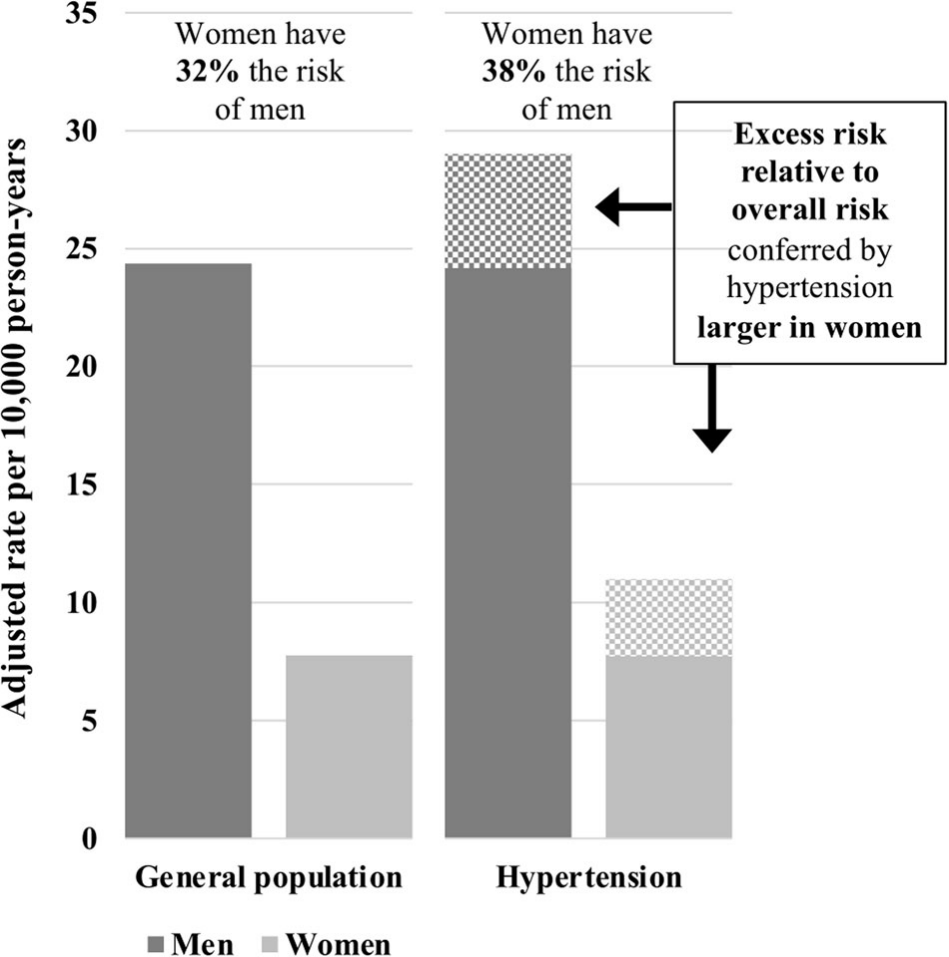
Adjusted incidence rate for myocardial infarction amongst people with stage 2 hypertension and overall rate. The study population was 471,988 people in the UK Biobank without a history of CVD followed up for 7 years [11]. Stage 2 hypertension was defined as systolic blood pressure ≥ 140 mmHg or diastolic blood pressure ≥ 90 mmHg.

The seminal Prospective Studies Collaboration showed that blood pressure was strongly linked to the risk of CVD, without evidence of a threshold down to at least 115/75 mmHg [8]. A meta-analysis conducted on data of 1,197,472 individuals (44% women, aged 19–107 years) from 124 prospective cohort studies found no evidence for a sex difference in the relationship between systolic blood pressure and either the risk of stroke or ischaemic heart disease [9]. Each 10-mmHg increment in SBP was associated with an approximately 25% higher risk of stroke and 15% higher risk of ischaemic heart disease, in both women and men. However, some studies have shown that hypertension does not confer the same risk in women and men [6]. For example, a study on 27,542 individuals (54% women) from the United States found that an increase in CVD risk began at a lower SBP in women than men [10]. Indeed, the observed increase in CVD risk in women at a SBP level of 100–109 mmHg relative to SBP < 100 mmHg was equal to the increase in risk in men at a SBP level of 130–139 mmHg. Similarly, a study of 471,998 individuals from the UK Biobank found a stronger association between SBP and hypertension with myocardial infarction in women than men [11]. For instance, the women-to-men ratio of hazard ratios for elevated blood pressure (defined as SBP 120–129 mmHg and DBP > 80 mmHg) was 1.83 (95% confidence interval (CI) 1.33–2.52), indicating that the association of elevated blood pressure with relative MI risk was 83% stronger in women than men. For both stage 1 and stage 2 hypertension, the association was about 45% stronger in women than men. Figure 1 illustrates the excess risk of MI conferred by hypertension in women and men. While the absolute risk of MI remains lower in women than men, the female advantage in terms of MI risk decreases in the presence of hypertension. In the overall population, women have 32% of the risk of men (adjusted incidence rate per 10,000 person-years 7.76 events in women versus 24.35 events in men), while in individuals with hypertension, women have 38% the risk of men (adjusted incidence rate per 10,000 person-years 11.18 events in women versus 28.92 events in men). This is because the excess risk conferred by having hypertension (the dotted area in Fig. 1) relative to the overall risk (the solid area in Fig. 1) is larger in women than men.

In addition to sex differences in cardiovascular risk factor associations, women and men often receive different treatments for the management of cardiovascular risk factors, and observational studies suggest a different optimal dosage of cardiovascular drugs for women and men [12–14]. Even though some treatment differences might be biologically justified, current guidelines do not differentiate per sex [15]. Evidence from trials about sex differences in the effects of medical interventions is lacking and would ultimately be needed to reach concordance between clinical practice with guidelines. Yet, females remain underrepresented in clinical trials and sex-specific results are often not reported [16–18]. This is problematic because women and men have an equal lifetime risk of CVD, have a different pathophysiology and because women with CVD have worse outcomes than men [19]. As a result, trials are not generalisable and underpowered to draw conclusions about the effect of drugs in women.

The above evidence on potential sex differences in the strength of the relationship between high blood pressure and CVD comes from observational studies. While invaluable, these non-randomised studies are subject to several biases, such as reverse causation, residual confounding, and unmeasured confounding [20, 21]. Therefore, associations found in observational studies may not reflect casual relationships. As such, it is unclear whether the sex differences in the magnitude of the association between high blood pressure and CVD are related to inherent biological differences between women and men. Mendelian randomisation (MR), a method for causal inference using genetic variants as instrumental variables, will be proposed to assess causality. In this review, we elaborate on why and how sex-specific MR and drug target MR could be used to uncover whether sex differences in risk factor and drug target effects exist and what the anticipated on-target effects of a novel drug compound would be. Because hypertension is a major modifiable risk factor and it is amenable to MR studies, hypertension will be the main example discussed in this review.

### Why other approaches are needed to determine the causality of sex differences in risk factors

Questions about causal relationships are fundamental to many epidemiological studies. One needs to know cause and effect, and to differentiate between causation and correlation, in order to understand disease aetiology, to assess the impact of medical or public interventions, to advise clinical practice, and to guide drug development.

At population level, a common way of assessing causality is through ‘manipulation’ of the exposure variable; or as Holland stated in 1986, “no causation without manipulation” [22]. The idea is that manipulation of a variable, by means of an intervention of some kind, is necessary to investigate the causal effect of an exposure on an outcome. Subsequently, a causal effect is estimated as the change in outcome that results from setting the exposure variable to a different level. In line with this, randomised controlled trials (RCTs) are the gold standard to investigate whether the effects of treatments are causal. As individuals are randomly allocated at baseline to either an intervention or control group, the groups are comparable in their average baseline variables and potential confounding factors. If the randomisation and outcome assessment is done with blinding, any differences in outcomes can be attributed to the intervention.

Confounding and reverse causation can lead to differences between the observed association and causal effect of a risk factor or intervention. Confounding exists when a spurious association arises because of a variable that causes both the exposure and the outcome. For example, individuals who have a low blood pressure level are observed to have a lower risk of CVD. However, a confounding factor, physical activity, is a common predictor of both blood pressure and cardiovascular outcomes, so it might be that low physical activity rather than a high blood pressure level is the cause of better cardiovascular outcomes. Spurious associations may also arise due to reverse causation. For example, C-reactive protein (CRP) has been linked with CVD, however, inflammatory cytokines from atheromatous plaque or adipose tissue raise the level of CRP. So in this association, CRP is an indicator instead of the cause of an increased CVD risk.

Statistical techniques such as stratification and covariate adjustment aim to correct for biases, however, due to their non-randomised nature, bias can never be ruled out completely, and importantly, one typically does not have a benchmark available to determine when adjustment for these biases is sufficient [23]. Therefore, it is uncertain whether an observed sex difference in the association between a risk factor and an outcome is a causal effect or whether it is due to bias. Other study designs are helpful to investigate causal relationships. RCTs are the gold standard in question around causality, but can often not be performed because of practical, ethical, or financial reasons. An alternative approach that makes it possible to study causal hypotheses from observational data, is Mendelian randomisation.

### Overview of Mendelian randomisation

#### What is Mendelian randomisation?

Mendelian randomisation is a specific type of instrumental variable analysis, which utilises genetic variants strongly associated with a risk factor (e.g., high blood pressure) to estimate the causal effect between the risk factor and outcome [24, 25]. The robustness of MR stems from the fact that genes are fixed at gamete forming, preventing reverse causation, which similarly minimises the number of common causes (confounders) between genetic variants and any outcome. As such, MR has been referred to as nature’s randomised controlled trial, referring to the shared designed features between MR and RCTs.

#### Assumptions and concept of Mendelian randomisation

MR has three core assumptions. First, the genetic variant should be strongly associated with the risk factor of interest. This is known as the relevance assumption. Second, there should be no common cause between the genetic variant and a risk factor or outcome. This assumption, known as the independence assumption, usually holds in MR because one’s genotype is defined at gamete forming and is rarely changed by exposure. Third, the genetic variant should affect the outcome only through the risk factor, not via other pathways or direct pathways, referred to as the exclusion restriction or absence of horizontal pleiotropy.

**Fig. 2:**
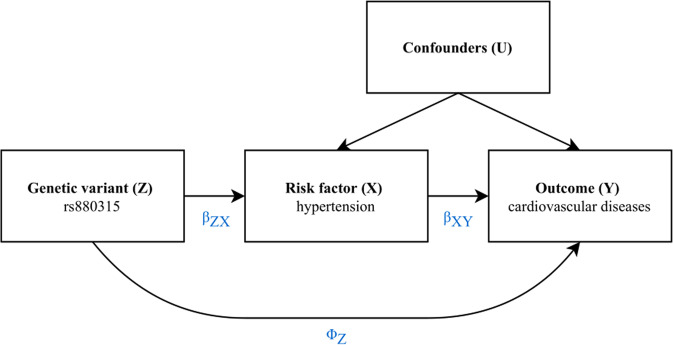
Diagram of the concept and assumptions for Mendelian randomisation. The presence of an arrow indicates a causal effect. The genetic variant Z is a valid instrument when it is strongly associated with the risk factor of interest X (relevance assumption) and when it has an effect on the outcome of interest Y exclusively through X, and not via another pathway or a direct pathway (absence of horizontal pleiotropy or exclusion restriction). So *Φ*_Z_ should be 0. This diagram uses the genetic variant rs880315 (located at chromosome 1, position 10796866) as an example, as it is known to be related to SBP and DBP [38].

If all assumptions are satisfied, any association of the genetic variant with the outcome is assumed to be through the variant’s association with the risk factor. This implies a causal effect of the risk factor on the outcome. A graphical representation of the concept and assumptions of MR is given in Fig. 2, where the second assumption is encoded by the absence of a common cause of the genetic variant, and the risk factor and outcome nodes.

When assumptions are met, the simplest way of estimating the causal effect of the risk factor on the outcome is by using the ratio of coefficients method [26]. As illustrated in Fig. 2, the direct effect of the instrumental variable Z on the outcome Y equals the product of effects underlying the path through the risk factor (*β*_*ZY*_ = *β*_*ZX*_ × *β*_*XY*_). From this, it follows that the causal effect of risk factor X on outcome Y is estimated by dividing the association of the instrumental variable on the outcome by the association of the instrumental variable on the risk factor: $\beta _{XY} = \frac{{\beta _{ZY}}}{{\beta _{ZX}}} = \frac{{\beta _{ZX} \times \beta _{XY}}}{{\beta _{ZX}}}$.

#### Comparison of Mendelian randomisation with randomised controlled trials

**Fig. 3:**
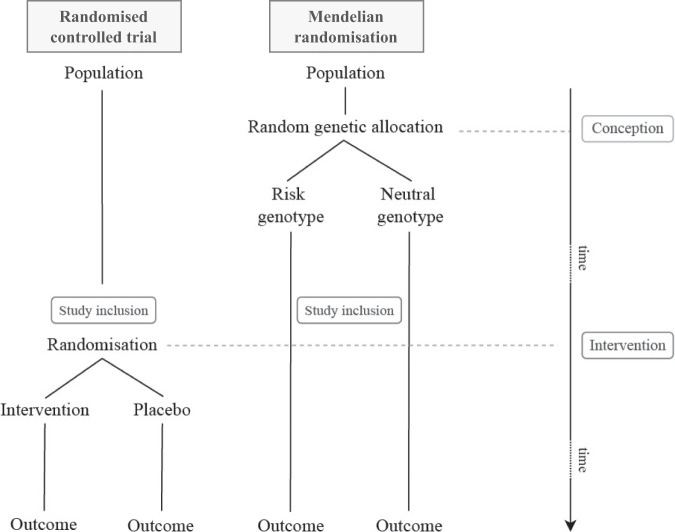
Comparison of a randomised controlled trial (RCT) design and a Mendelian randomisation (MR) study. In both a RCT and a MR study, randomisation allows for comparability of groups in their average baseline variables and potential confounding variables. Randomisation of individuals in RCTs takes place at a later point in life (and after study inclusion) than the random allocation of alleles at conception (before study inclusion) in MR studies. This makes MR studies more susceptible to survivor bias than RCTs.

The concept of MR can be illustrated by the similarity between a RCT and a MR design (Fig. 3). In RCTs, individuals are randomly allocated to treatment arms, and these groups will on average have comparable distributions of covariates, resulting in a comparable baseline risk of disease. In MR, this randomisation results from the fact that the SNPs that affect the risk factor are randomly allocated at gamete forming.

While a trial is a useful analogy, there are important differences. A fundamental difference is the moment of randomisation (Fig. 3). In RCTs individuals are enroled and soon after randomised to a treatment arm at a later point in life (sometimes at birth, but often years later). In MR studies, random allocation takes place at conception and participants are only included in a study after they have survived sufficiently long, for birth cohort this means until birth, but often enrolment occurs much later in life. This makes MR studies susceptible to survivor bias, a form of selection bias. Methods have been developed to address this issue in MR, although it cannot be ruled out completely [27].

#### Drug target Mendelian randomisation

**Fig. 4:**
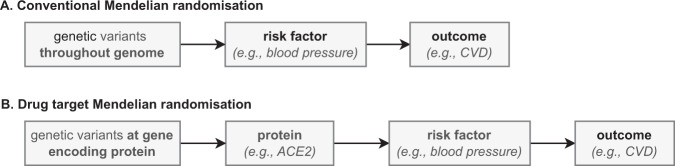
Comparison of conventional Mendelian randomisation and drug target Mendelian randomisation. **A** MR studies the effect of causal risk factors such as blood pressure on the risk of CVD by selecting genetic variants throughout the genome as instruments. **B** Drug target MR studies the on-target effect of protein or mRNA drug target perturbation on the risk of CVD by selecting genetic variants at or around the gene that encodes the protein.

Drug compounds may affect an outcome through on-target pathways (acting through perturbation of the intended drug target(s)) as well as through off-targets pathways (by perturbing pathways which side-step the intended drug target(s)). MR can be used to evaluate the on-target effect of protein or mRNA drug target perturbation, which may be relevant for de novo drug development or as a starting point for drug repurposing [28, 29]. In drug target MR, the instrumental variable is a selection of one or more SNPs that are in or around the drug target encoding gene (Fig. 4). Drug target MR can contribute to evidence on which targets should be pursued in drug development. It is a way to study what effects protein perturbation on disease might have.

While RCTs study the effects of a specific drug compound on health outcomes, drug target MR attempts to understand the role of the drug target (i.e. the encoded protein) in a disease. Therefore, drug target MR studies are unlikely to replace RCTs for testing drug efficacy. However, drug target MR studies may reduce costs in the development of drugs through early identification of targets that might not elicit the expected effects. Genetic evidence can also help clarify the causal mechanisms behind therapeutic efficacy or safety of drugs that have already received approval.

#### Sex-specific Mendelian randomisation

Sex-combined MR could mask potential sex differences, either in direction or magnitude, in the causal effects of risk factors and drug targets. The basic principles of MR can easily be extended to MR aimed at establishing sex-specific risk factor or drug target effects. In sex-specific MR, genetically determined relationships between a risk factor and outcome are evaluated separately for women and men. The basic principles of sex-specific MR can also be extended to sex-specific drug target MR. A previous study showed that 30% of genetic co-expression is influenced by sex and that this is often the case for targets of FDA-approved drugs [30]. Sex-specific drug target MR can therefore potentially identify sex-specific drug target effects.

Ideally, instruments are selected from a sex-stratified genome wide association study (GWAS) for each sex separately and are used as SNP-exposure effects in the MR analyses. Recent analyses of the UK Biobank found that 61 of 84 (72.62%) nonbinary and 42 of 446 (9.42%) binary traits had at least one autosomal genetic variant with a significantly different effect in women than in men (at a *p* < 1 × 10^–8^ threshold) [31]. Also, a GWAS on type 2 diabetes found seven novel variants to be associated with the trait, of which one was male-specific, and one was female-specific [32]. This shows that the number of selected instruments, the selection of instruments itself and the weights need not be the same in subgroups. This is not necessarily a limitation and does not change the interpretation of findings of MR studies. In MR (or any instrumental variable analysis) the interest is in the non-genetic effects (e.g., from SBP on CVD), which should be distinguished from an interest in the genetic effect (e.g., genetic effects on SBP). MR uses genetic instruments and sex differences in the genetic associations with SBP (or any other exposure) do not imply that the SBP effect on an outcome will be different between men and women. In the same way, a different effect between men and women of SBP on CVD does not imply that the genetic effects on SBP are different between sexes.

When no sex-stratified GWAS data is available, analysis could be conducted on aggregate data. However, if there is considerable heterogeneity between the genetic effects between men and women (on SBP) this will possibly invalidate any MR analysis using sex-combined GWAS estimates with the exposure of interest. Since sex-specific MR studies rely on sex-stratified GWAS results, it is of importance to conduct more sex-specific GWAS and to have sex-specific GWAS summary statistics publicly available.

When exploring subgroup-specific effects, sample size is often limited compared to analyses in the overall sample, which reduces power and may require lowering the *p*-value threshold or different linkage disequilibrium (LD) clumping parameters to ensure there are sufficient instruments to conduct a MR analysis. While MR principles merely require genetic instruments to be strongly associated with the exposure, and do not call for any specific cut-off (e.g., such as the GWAS significant threshold of 5 × 10^–8^), a lower *p*-value threshold (or equivalently lower F-statistic or explained variance) increases the risk of weak-instrument bias. In one-sample settings (where the genetic association with the exposure and outcome are estimated in the same data), this bias acts towards the direction of the (potentially) confounded observational association between exposure and outcome, where the bias increases with weaker instrument strengths [33]. As a result, interpretation of results is less straightforward.

Another challenge arises when sex-specific genome-wide significant SNPs are not available for both sexes. For example, sex-stratified GWASs found multiple genetic variants associated with the waist-to-hip ratio (WHR) and waist circumference in women, but no significant variants were found in men [34]. In such case, it is questionable how a men-specific MR should be performed. An option would be to use less strict *p*-value thresholds for statistical significance or different LD clumping parameters in men-specific GWAS, although care should be taken that there are sufficiently strong instruments in both men and women.

In typical GWAS, only SNPs on the autosomal chromosomes are evaluated. This could affect the results of an MR study, especially when valid instruments to proxy risk factor effects are the SNPs on the sex chromosomes.

#### Examples of sex-specific Mendelian randomisation studies

Despite potential problems and challenges in sex-specific MR studies, previous sex-specific MR studies have been performed. These demonstrate that MR can explore sex-specific relationships that have not been studied yet, or that MR can either confirm or refute existing observational evidence on sex differences in risk factor effects on disease. The first study that used sex-specific MR found a sex difference in the effect of BMI on the risk of type 2 diabetes, and in the effect of waist-related traits on chronic obstructive pulmonary disease and renal failure [35]. Sex-specific genetic risk scores (GRSs) were calculated using the primary genome-wide significant SNPs from a men-only, women-only, and combined-sexes analysis. Variants were weighted by sex-specific associations. The estimates were replicated when using a variety of SNP-selection and weighting approaches, such as combined-sexes primary SNPs and combined weights.

Another sex-specific MR study based on data from the UK Biobank found no sex difference for the strength of the causal effect of genetic liability to type 2 diabetes on the risk of CHD [36]. This was in contrast with strong evidence from observational studies that consistently found evidence for a stronger association in women than men. The MR study used a selection of 270 SNPs from the sex-combined European DIAMANTE GWAS, and sex-specific weights obtained from the sex-specific European DIAMANTE GWAS.

As a final example, a sex-specific MR studied the effect of genetically predicted sex hormone binding globulin (SHBG) on ischaemic heart disease, using a sex-specific SNP-selection [37]. From classical epidemiological studies, men are observed to have a lower risk of CVD at a lower serum SHBG level (independent from their testosterone level), while this association was observed to be absent or in the opposite direction for women. The MR study found genetically proxied SHBG to be negatively associated in men (OR 0.78 per standard deviation, 95% confidence interval (CI) 0.70–0.87), which is contrary to the findings from previous observational studies. There was no evidence for a sex difference in the effect for women (OR 0.89 per standard deviation, 95% confidence interval (CI) 0.74–1.08, test for sex difference, *p*-value of 0.32).

### Spotlight on hypertension

#### GWAS for blood pressure traits

The largest genetic association study of blood pressure traits performed a meta-analysis on a sample of in total 757,601 individuals of European ancestry. This sample was drawn from the UK Biobank cohort and data obtained by the International Consortium for Blood Pressure (ICBP) [38]. The UK Biobank sample consists of 458,577 individuals of European ancestry (after performing quality checks), from which 54.2% were women, and the ICBP collected data on 299,024 European ancestry individuals from 77 different cohorts, from which 51% were women. This study identified over 1000 independent signals at 901 loci for systolic blood pressure (SBP), diastolic blood pressure (DBP) and pulse pressure, of which many loci were novel as compared to previous genetic association studies [39]. No sex differences were reported.

#### Mendelian randomisation evidence for hypertension as risk factor for CVD

A recent MR study found a strong relationship of both genetically proxied SBP and DBP with cardiovascular outcomes. The study used 253 SNPs to proxy the effect of blood pressure on incident coronary artery disease, stroke, and the combined outcome of CVD [40]. The variants were selected and weighted based on their association with SBP and DBP according to the GWAS from the ICBP. For every 10-mmHg increase of genetically proxied SBP, the risk of incident CVD increased by 49% (HR 1.49, 95% confidence interval (CI) 1.38–1.61). Similar results were found for coronary artery disease (HR 1.50, 95% confidence interval (CI) 1.38 to 1.63) and stroke (HR 1.44, 95% confidence interval (CI) 1.22 to 1.70). Subgroup analyses by sex showed similar shapes of the relationship between genetically proxied blood pressure and CVD in women and men. However, analyses were performed using GRSs that used the same weights and the same selection of SNPs for both sexes, and sex differences were not formally quantified.

#### Drug target Mendelian randomisation evidence on antihypertensive drugs

Recently, a drug target MR study was performed to study the effects of several antihypertensives on the risk of coronary heart disease and stroke [41]. The selected genetic variants were associated with SBP and were in the loci of the genes coding for the corresponding targets of angiotensin-converting enzyme (ACE) inhibitors, beta-blockers, and calcium channel blockers. For example, the *ACE* gene at chromosome 17 was selected to proxy the effect of ACE. A significant protective effect was found on stroke for the ACE inhibitors (RR = 0.21, 95% confidence interval (CI) 0.06–0.72). Such results strengthen trial evidence, because similar results are found under different assumptions, also known as triangulation [42, 43].

Previous observational studies have shown heterogenous effects of the *ACE* locus on blood pressure in men versus women. It was suggested that *ACE*, or a nearby gene, is a candidate gene for hypertension in men, but not in women [44]. Also, women had lower ACE activities than men, even when maximum inhibition of ACE activity was attained [45]. Sex-specific MR studies allow to test for sex differences in the causal effects of the *ACE* locus. This would add evidence on potential sex differences in the effect of drugs targeting the *ACE* gene.

An increasing number of observational studies suggest sex differences in the pathophysiology of hypertension and pharmacogenetics regarding blood pressure lowering drugs [46]. While several sex-specific MR studies on risk factors have been conducted in the past years, literature on sex-specific drug target MR studies is lacking. Conducting a drug target MR for women and men separately provides an indication of sex difference in drug target effects as drug compounds may have different associations in each sex. Subsequently, such results could be further explored by conducting sex-stratified drug trials. If evidence for sex differences in drug effects is found, clinical guidelines should provide sex-specific treatment recommendations, which could eventually optimise treatment and health outcomes in both women and men.

## Conclusion

Mendelian randomisation is a valuable method to assess causal effects of risk factors on disease outcomes. Sex-specific MR, where instrumental variable selection and construction of GRS is preferably done for each sex separately, explores sex differences in causal risk factor and drug target effects, and answers the question of whether observed sex difference in the strength of the association between hypertension and cardiovascular disease are causal. Results from MR analyses can guide sex-specific recommendations in guidelines. Drug target MR could contribute to study drug-target effects, which could result in improved health outcomes in both sexes.
